# Microwave-assisted pyrolysis of *Pachira aquatica* leaves as a catalyst for the oxygen reduction reaction

**DOI:** 10.1039/d0ra01078b

**Published:** 2020-03-20

**Authors:** Sun-Tang Chang, Huan-Ping Jhong, Yu-Chung Chang, Chia-Chi Liu, Tai-Chin Chiang, Hsin-Chih Huang, Chen-Hao Wang

**Affiliations:** Department of Materials Science and Engineering, National Taiwan University of Science and Technology Taipei 10607 Taiwan chwang@mail.ntust.edu.tw; Global Development Engineering Program, National Taiwan University of Science and Technology Taipei 10607 Taiwan hchuang@mail.ntust.edu.tw

## Abstract

In this study, biomimetic Mg–N_*x*_–C_*y*_ from *Pachira aquatica* leaves were mixed with carbon black (L/C catalyst), in which the mixture was treated by a conventional microwave oven at 700 W and 2 min, exhibiting high catalytic activity for the oxygen reduction reaction (ORR). By using a microwave-assisted process, it not only offers a cheaper and faster way to synthesize the catalyst compared to the conventional furnace process but also avoids the decomposition of the N_4_-structure. Using the optimized conditions, the L/C catalyst exhibits an electron transfer number of 3.90 and an HO_2_^−^ yield of only 5% at 0.25 V *vs.* RHE, which is close to the perfect four electron-transfer pathway. Besides, the L/C catalyst offers superior performance and long-term stability up to 20 000 s. The L/C catalyst contains a high proportion of quaternary-type nitrogen, Mg–N_*x*_–C_*y*_, and –C–S–C– which can be the active sites for the ORR.

## Abstract 2

In this study, biomimetic Mg–N_*x*_–C_*y*_ from *Pachira aquatica* leaves were mixed with carbon black, in which the mixture was treated by a conventional microwave oven at 700 W and 2 min, exhibiting high catalytic activity for the oxygen reduction reaction.

## Graphical abstract

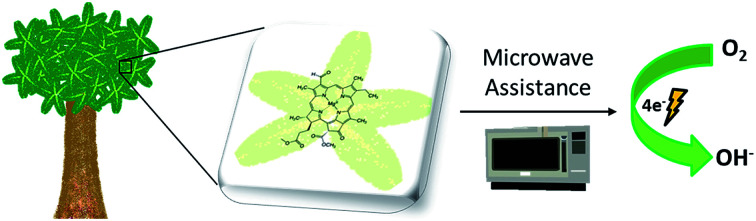

## Introduction

Fuel cells, and especially proton exchange membrane fuel cells (PEMFCs) and alkaline anion exchange membrane fuel cells (AAEMFCs), are widely utilized in stationary power plants and transportation power sources. The only product of fuel cells is water, so fuel cells are “eco-friendly” and provide “green energy”. However, fuel cells are expensive because the used catalysts need a large amount of Pt and Pt-alloy. Furthermore, the sluggish kinetics of the oxygen reduction reaction (ORR) at the cathode greatly limits the energy conversion efficiency of fuel cells.^1–6^ In 1964, Jasinski *et al.* became the first group to report on the use of a non-precious metal catalyst for the ORR.^7^ In the subsequent 40 years, more scientists have started to investigate and discover new non-precious metal catalysts.^8–12^ In the last decade, Dodelet's group had developed a metal–organic-framework-derived electrocatalyst for the ORR, which exhibited a high power performance.^13,14^ With the improved mass-transfer properties, the volumetric activity is further enhanced. Zelenay's group had developed non-precious metal catalysts of the ORR derived from polyaniline–metal-carbon with long-term stability and high activity.^15,16^ The uniform distribution of nitrogen sites and the presence of the graphitized carbon phase play a role in activity for the ORR. Additionally, due to the electron density of the low-symmetry N_4_ structure being high, it may interact with O_2_ easily and strongly. These kinds of low-symmetry N_4_ structures have been utilized as non-precious metal catalysts with high output performance,^17–19^ as well.

Recently, transition metal-based compounds, such as oxides, sulfides, and nitrides, have been used as catalysts or supports for electrocatalytic applications.^20–22^ Dai's group examined cobalt oxide-based nanocrystals on graphene or carbon nanotubes as non-precious metal catalysts in alkaline media.^23–25^ With the strongly coupled property, the electrocatalysts exhibited good ORR activity. Wang *et al.* used Fe^3+^ doped Co_3_O_4_ to form the octahedral Co–Fe spinel oxides and grew it on N-doped carbon nanotubes as a bifunctional electrode for zinc–air batteries.^26^ According to the structural characterization and theoretical calculation, the obtained catalyst had the effect of spin and charge, resulting in the enhanced oxygen catalytic activity. Additionally, transition metal catalyst-based materials as a non-precious metal catalyst in AAEMFC have been developed in our previous studies. With the effects of the nanostructure, heteroatoms doping, and surface property, the non-precious metal catalysts showed remarkable ORR activity.^27–31^ However, there are lots of preparation methods involved in toxic solvents. Waste biomass contains a high amount of carbon, oxygen and nitrogen elements, and it can be used as an inexpensive precursor of carbon to lower down the environmental impact of the procedure. Natural leaves are one of the biomass-derived materials which contain the inherent chlorophyll; the central structure is Mg–N_4_, one of the N_4_-macrocyclic structures. Hence, scientists try to use waste leaves as the non-precious metal catalysts for ORR. Ginkgo leaves were used as precursors to fabricate non-precious metal catalysts by the pyrolysis and acid leaching; Pan *et al.* were synthesized nitrogen-doped porous carbon nanosheets by carbonization of ginkgo leaves and then followed by ammonia post-treatment which exhibited high surface area and excellent ORR performance;^32^ Gao *et al.* presented nitrogen-doped carbon shell structure for ORR and the experimental result was consistent with the quantum mechanics calculations. Additionally, the fullerene-like carbon shell was also used for lithium-ion batteries;^33^ Razmjooei *et al.* prepared a porous network carbon catalyst with multi heteroatom-doping, not only yellowish but also greenish ginkgo leaves were compared the activity of ORR. Based on the original property, the yellow leaves could maintain a more stable structure than the green one, resulting in the superior surface properties and catalytic activity.^34^ On the other hand, Gao *et al.* also used amaranthus waste to synthesize nitrogen-doped carbon catalyst, which showed superior surface area, conductivity, and ORR performance.^35^ London plane leaves,^36^ bamboo leaves,^37^ and *Euonymus japonicus* leaves^38^ were also used to prepare nitrogen-doped porous carbons as electrocatalysts for ORR.

In this work, *Pachira aquatica* leaves are utilized as precursors, because the inherent chlorophyll is known to possess an N_4_-macrocyclic structure. To overcome the time-consuming of the traditional furnace for pyrolysis, a microwave oven replaces the traditional furnace to pyrolyze the leaves quickly without decomposing the N_4_-macrocyclic structure. The microwave oven has the advantages of non-toxic, fast, simple, and cheap which is beneficial to the process. Also, *Pachira aquatica* leaves are taken from natural and zero cost, which are biomass-derived eco-friendly catalysts for the fuel cell.

## Experimental

### Preparation of catalysts

*Pachira aquatica* leaves (the courtyard of our department) and carbon black (XC-72R) mixtures were immersed in 10 mL ethanol (96%, technical, Acros) and agitated for 30 min using an ultrasonicator. This is followed by removing the solvent using a rotary evaporator (Eyela, N1100). The mixture was put in a crucible, which was then placed in a conventional microwave oven (Panasonic, ST557). The powers of the microwave oven and treatment times were varied as required. The powers were set to the medium value of 700 W and a medium-low value of 400 W.

To prepare the pyrolyzed leaves that were mixed with carbon support (L/C catalyst), the leaves were washed several times using DI water and introduced into a juicer machine. 300 mg of mashed leaves were mixed with 200 mg of XC-72R and 10 mL of ethanol, to form L/C mash. Rotary evaporator removed the residual solvent from the L/C mash. The dry L/C mash was then placed in the microwave oven on two powers, 400 W and 700 W, for three treatment times, 1, 2, and 3 min, to yield the L/C catalysts. The L/C catalysts are denoted as L/C catalyst-700 W-1 min, L/C catalyst-700 W-2 min, L/C catalyst-700 W-3 min, L/C catalyst-400 W-1 min, and L/C catalyst-400 W-2 min.

### X-ray diffraction (XRD)

High-resolution X-ray diffraction (XRD) spectra were obtained at room temperature using beamline 01C2 (at the National Synchrotron Radiation Research Center, Hsinchu, Taiwan) with Mo Kα1 radiation (*λ* = 0.70930 Å) and an energy of 25 keV in a limited range of angles. The 2*θ* scans were carried out at a scan rate of 10° per minute, in steps of 0.05° from 5° to 45°. All XRD spectra in this work were referenced to Cu Kα1 radiation (*λ* = 1.54056 Å).

### Raman spectroscopy

Raman spectroscopy, a He–Ne laser (wavelength: 632.8 nm, Jobin-Yvon LabRAM HR800-Confocal micro-Raman spectroscopy) as the excitation source, was carried out to identify the D- and G-peaks.

### X-ray photoelectron spectroscopy (XPS)

The XPS were obtained at an end station at NSRRC beamline BL24A1. The photoemission spectra were collected using an analyzer (SPECS PHOIBOS 150). The grating enabled the photon energy to vary from 240 to 840 eV, with a substantial proportion of high-order harmonics. All XPS spectra were obtained using photons with an energy of 700 eV.

### Electrochemical measurements and preparation of catalyst ink

Electrochemical measurements of a three-compartment cell were made using a potentiostat/galvanostat instrument (Biologic Bi-stat). The counter electrode and the reference electrode were a piece of Pt foil and a saturated calomel electrode (0.242 V *vs.* NHE), respectively. The working electrode was a rotating-ring disk electrode (RRDE, PINE AFE7R9GCPT) comprised of glassy carbon (GC) disk and a platinum ring. Here, the area of GC disk is 0.238 cm^2^ and the collection efficiency (*N*) of the ring is 0.383. All potentials in this work were referenced to the reversible hydrogen electrode (RHE). An oxygen-saturated 0.1 M KOH solution was used as the electrolyte in the ORR test.

L/C catalyst ink was prepared by mixing 80 mg of L/C catalyst with 20 mL deionized water. 20 μL of this L/C catalyst ink and 5 μL of 0.1 wt% FAA ionomer (fumion® FAA-3 solution in NMP, FuMA-Tech) were dropped onto the GC disk, which was then left to dry in air at room temperature. Linear scan voltammetry (LSV) was applied to carry out on the catalysts for the ORR. The ORR curves at the GC disk were obtained at a low scan rate of 10 mV s^−1^ to reduce the otherwise substantial non-faradaic current to be generated by the catalysts. To determine the yield of HO_2_^−^ in the ORR that was produced on the GC disk, 1.2 V was applied to the ring to generate a current that oxidized the HO_2_^−^. For comparison, 20 wt% Pt/C was prepared by dispersing 20 mg of Pt/C in 20 mL of DI water to form a Pt/C catalyst ink. 20 μL of thus Pt/C catalyst ink and 5 μL of 0.1 wt% FAA ionomer were dropped onto the GC disk, which was then left to dry in air at room temperature.

## Results and discussion

The pathway of the ORR in alkaline media is as follows.Reaction 1: O_2_ + 2H_2_O + 4e^−^ → 4OH^−^ *E*^0^ = 0.401 VReaction 2: O_2_ + H_2_O + 2e^−^ → HO_2_^−^ + OH^−^ *E*^0^ = −0.065 VReaction 3: HO_2_^−^ + H_2_O + 2e^−^ → 3OH^−^ *E*^0^ = 0.867 V

**Fig. 1:**
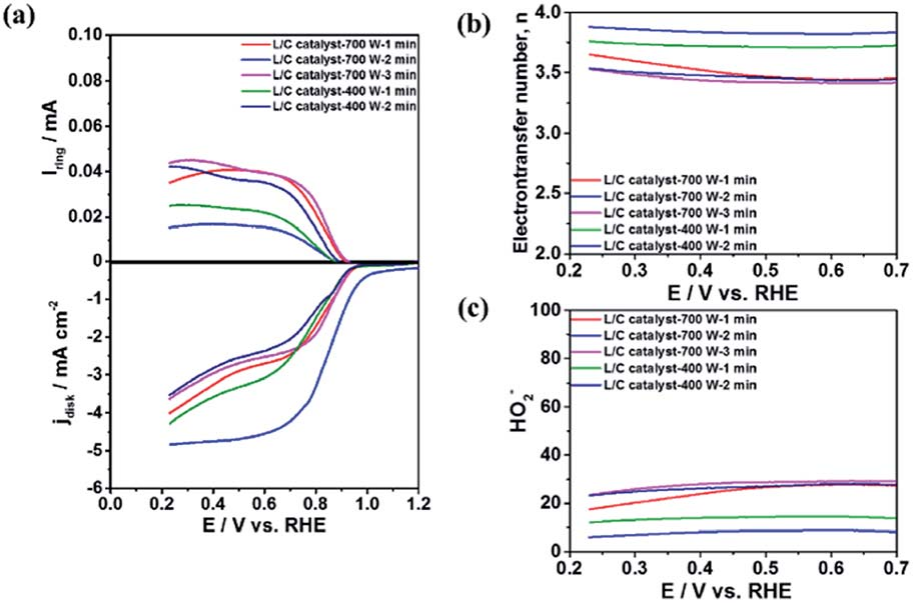
(a) The ORR curves; (b) electron transfer number; (c) HO_2_^−^ the yield of L/C catalysts with various powers and times. Scan rate: 10 mV s^−1^; rotation speed: 1600 rpm.

Reaction 1 represents a four-electron transfer, which proceeds by a direct pathway, in which oxygen is directly reduced to water. Reaction 2 proceeds by a two-electron transfer pathway, which allows the formation of an HO_2_^−^ intermediate, and chemically decomposed or electrochemically reduced to peroxide ions by Reaction 3. Fig. 1a plots the ORR curves of the various L/C catalysts using the RRDE method. The lower part plots the disk current density (*I*_d_) while the upper part plots the ring current (*I*_r_). To verify the ORR activity of the catalyst, the electron transfer number (*n* value) and the HO_2_^−^ yield (% HO_2_^−^) are calculated using eqn (1) and (2), respectively,1
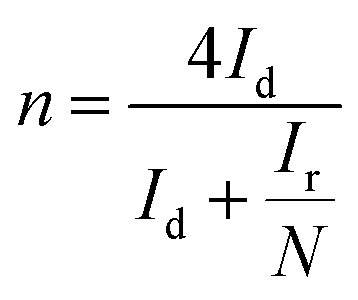
2
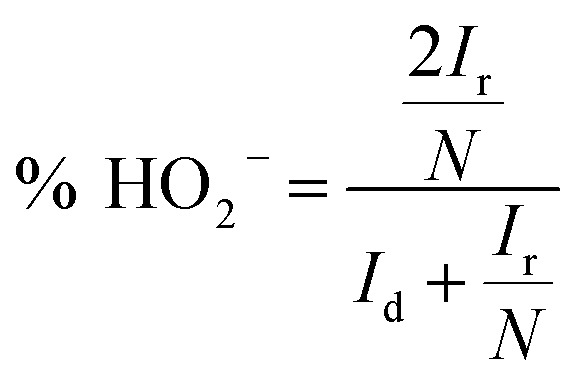
where *N* is the RRDE collection efficiency. Fig. 1b and c are the electron transfer numbers and the HO_2_^−^ yields *versus* the applied disk potentials, respectively, for various catalysts. Fig. 1a demonstrates that L/C catalyst-700 W-2 min exhibits the highest disk limiting current density of 5.5 mA cm^−2^ and the lowest ring current of 0.015 mA at 0.25 V among the catalysts with various treatment parameters. From Fig. 1b and c, its electron transfer number is 3.90 and the OH_2_^−^ yield is only 5.0%. L/C catalyst-400 W-1 min exhibits the second-highest ORR activity with the disk limiting current density of 4.5 mA cm^−2^, the electron transfer number of 3.75 and the HO_2_^−^ yield of 12.5%. The electron transfer numbers of L/C catalyst-700 W-1 min, L/C catalyst-400 W-2 min and L/C catalyst-700 W-3 min are 3.60, 3.52 and 3.50, respectively, and the corresponding HO_2_^−^ yields are 20.0%, 24.0% and 25.0% respectively. Raman spectroscopy, XRD, and XPS analyses are carried out to determine why L/C catalyst-700 W-2 min exhibits the highest ORR activity compared with other catalysts.

**Fig. 2:**
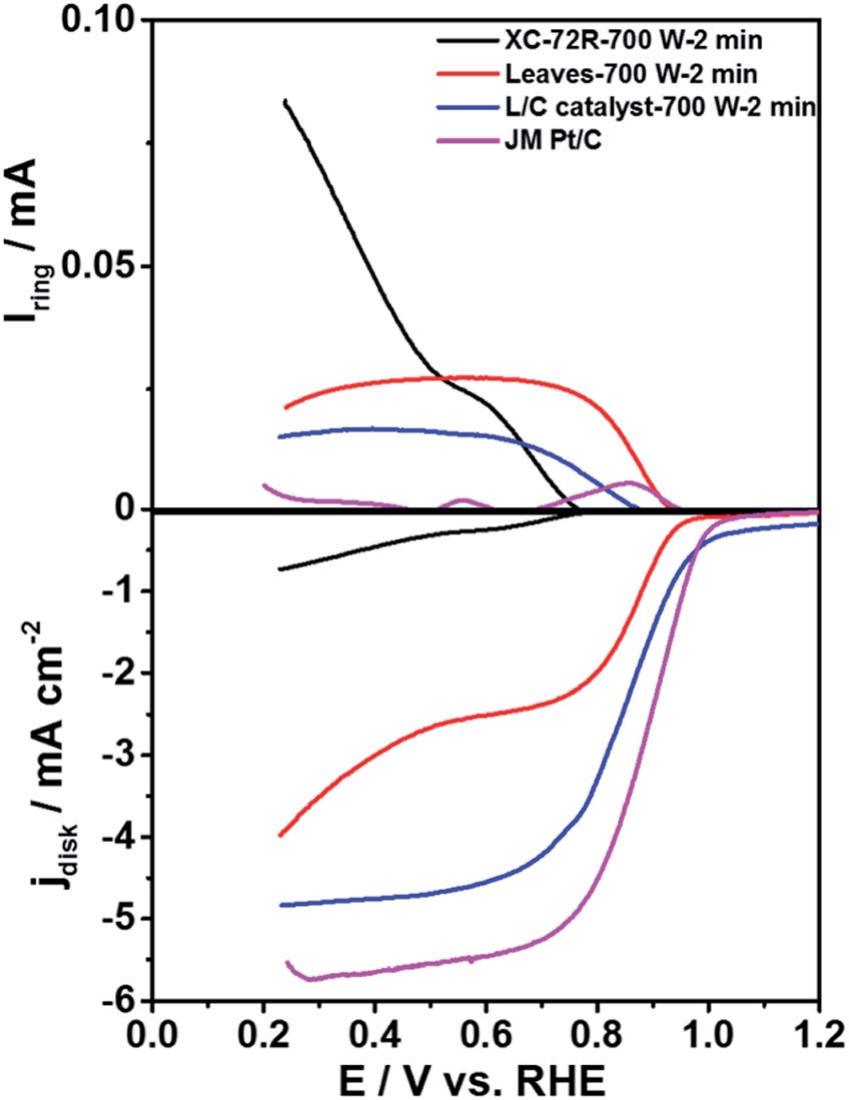
The ORR curves of various catalysts in 0.1 M KOH. Scan rate: 10 mV s^−1^; rotation speed: 1600 rpm.

To compare the ORR activity of L/C catalysts, Fig. 2 plots the ORR curves of Pt/C, carbon black that had been treated with 700 W for 2 min (XC-72R-700 W 2 min), pristine leaves that had been treated by 700 W for 2 min (Leaves-700 W-2 min) and L/C catalyst-700 W-2 min. Table 1 lists the calculated electron transfer numbers and the corresponding HO_2_^−^ yields of various catalysts from Fig. 2. The ORR activity decreases in the order of Pt/C, L/C catalyst-700 W-2 min, Leaves-700 W-2 min, and XC-72R-700 W-2 min, with electron-transfer numbers of 3.99, 3.90, 3.77, and 2.21, respectively, revealing that carbon black improves the ORR activity of the L/C catalyst because it improves the conductivity.

**Table 1:** The electron transfer numbers and the corresponding HO_2_^−^ yields of various catalysts calculated from Fig. 2

Samples	Electron transfer numbers	HO_2_^−^ yields
XC-72R-700 W-2 min	2.21	89.5%
Leaves-700 W-2 min	3.77	11.5%
L/C catalyst-700 W-2 min	3.90	5.0%
JM Pt/C	3.99	0.5%

**Fig. 3:**
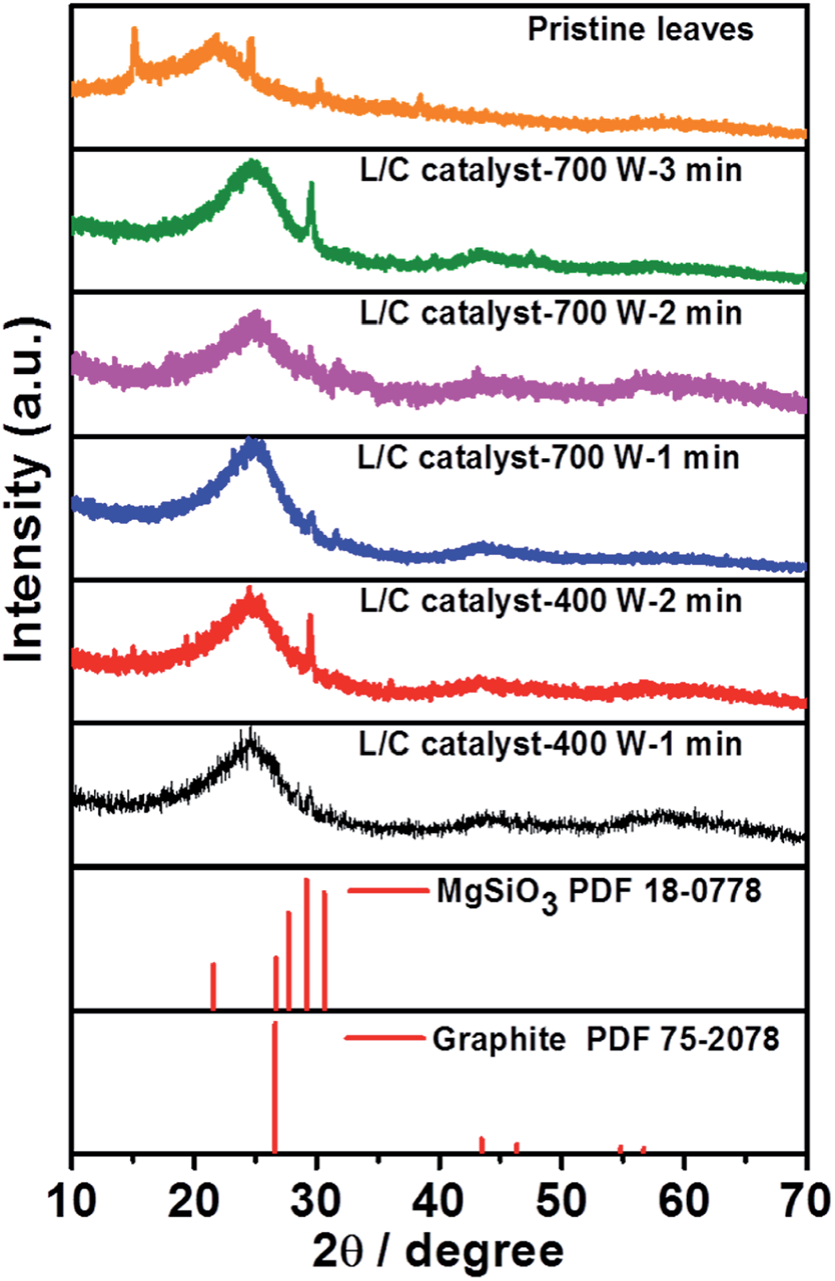
The XRD patterns of L/C catalysts with various microwave powers and times.

Fig. 3 plots the XRD results for the L/C catalysts. The XRD pattern of the pristine leaves is similar to that obtained by the works of Bajpai and Jain.^39^ According to their description, the chemical composition of the green tea leaves majorly consists of cellulose, lignin and structure proteins, insoluble proteins, and polyphenols. Several peaks are corresponding to its which locate at approximately 15.0°, 21.9°, 24.4°, 30.0°, and 37.9°, respectively. Moreover, the peak at 29.1° is associated with MgSiO_3_ and that at 26.4° is associated with the carbon material. When increasing the pyrolysis time, the MgSiO_3_ peak becomes stronger. It only shows the strongest peak of (221) plane at 29.1° without the peaks of (0–21) plane and (−201) plane at 27.7° and 30.6°, respectively. This might be due to the preferential orientation of the catalyst. The peak of graphite slightly downshifts to 26.7°, which can be associated with the increase of the vertical and lateral sizes of graphitic crystallites, as determined by Ungar *et al.*^40^Table 2 presents the intensity ratio of MgSiO_3_ to C. The microwave power of 700 W yields *I*_MgSiO_3__/*I*_C_ ratios of 1.18, 1.13, and 1.99 after 1, 2, and 3 min, respectively. The microwave power of 400 W yields *I*_MgSiO_3__/*I*_C_ratios of 1.16 and 1.53 after 1 min and 2 min, respectively. MgSiO_3_ is a kind of minerals that has a lower conductivity than carbon, so L/C catalyst-700 W-3 min and L/C catalyst-400 W-2 min have a high *I*_MgSiO_3__/*I*_C_ ratio, and exhibit poor ORR activities. However, this result is insufficient to explain why the best ORR activity in 700 W-2 min. Thus, Raman spectra and XPS spectra provide more information.

**Table 2:** The *I*_MgSiO_3__/*I*_C_ ratios determined from Fig. 3

Samples	*I*_MgSiO_3__/*I*_C_
L/C catalyst-700 W-1 min	1.18
L/C catalyst-700 W-2 min	1.13
L/C catalyst-700 W-3 min	1.99
L/C catalyst-400 W-1 min	1.16
L/C catalyst-400 W-2 min	1.53

**Fig. 4:**
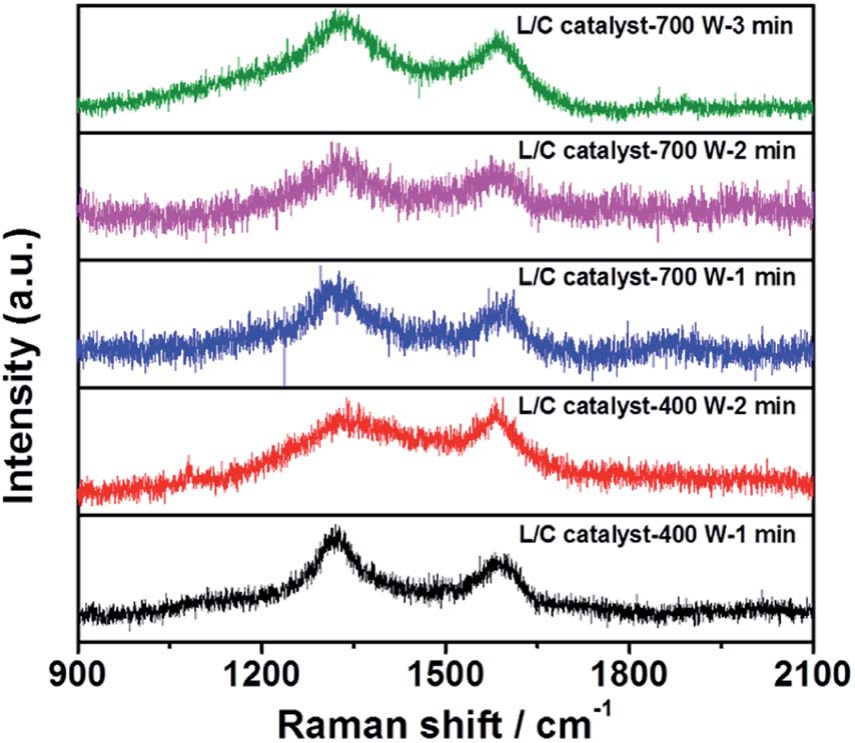
Raman spectra of L/C catalysts with various microwave powers and times.

The Raman spectra of all samples in Fig. 4 include two peaks at 1350 cm^−1^ and 1580 cm^−1^, which are the D-peak and the G-peak, respectively. The D-peak arises from the disordered structure and, in particular, the disorder of the sp^2^ hybridized carbon system in a carbon-based material. The high ratio of the D-peak reflects the poor electrical conductivity. The G-peak is associated with the E_2g_ mode in carbon-based materials, arising from the stretching of the C–C bond, which involves sp^3^ stretching. A high *I*_G_/*I*_D_ ratio is associated with the high electrical conductivity of the catalyst. Table 3 presents the *I*_G_/*I*_D_ ratios of all samples: L/C catalyst-700 W-2 min has the highest *I*_G_/*I*_D_ ratio, followed by L/C catalyst-400 W-1 min and others. Higher ORR activity seems to be associated with a higher *I*_G_/*I*_D_ ratio among the L/C catalysts.

**Table 3:** The intensity ratio of G-peak to D-peak calculated from Fig. 4

Samples	*I*_G_/*I*_D_
L/C catalyst-700 W-1 min	0.59
L/C catalyst-700 W-2 min	0.67
L/C catalyst-700 W-3 min	0.32
L/C catalyst-400 W-1 min	0.61
L/C catalyst-400 W-2 min	0.48

**Fig. 5:**
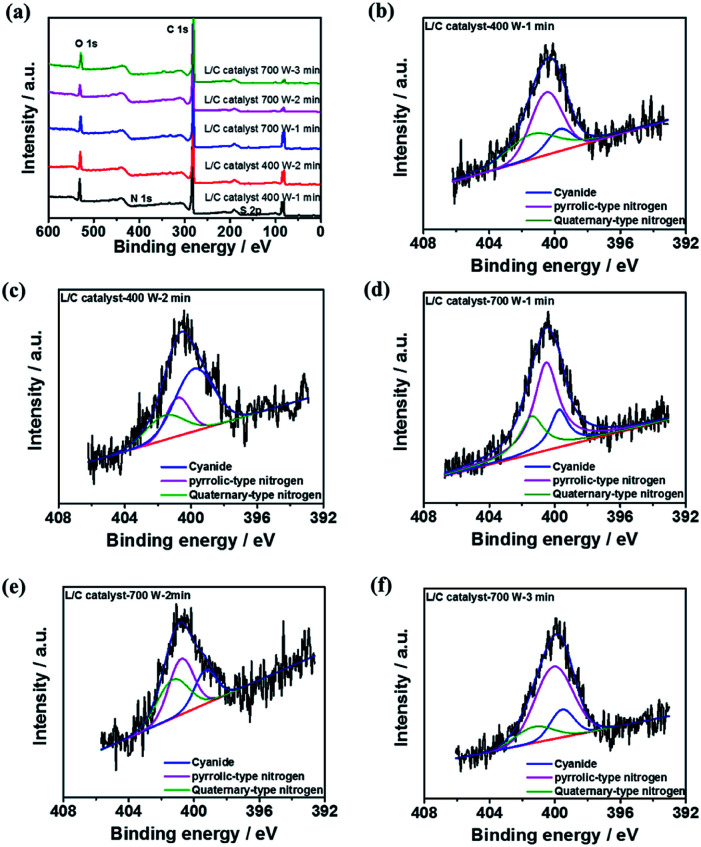
The XPS results of (a) all samples' survey pattern; N 1s spectra of L/C catalysts at: (b) 400 W-1 min, (c) 400 W-2 min, (d) 700 W-1 min, (e) 700 W-2 min, (f) 700 W-3 min.

XPS spectra are obtained to elucidate the chemical compositions of L/C catalysts. All samples contain six main elements, which were O, C, Si, Mg, N, and S, as shown in the survey pattern of Fig. 5a. According to other investigations, N and S, when co-doped in catalysts, can provide active sites for the ORR.^41–43^ In L/C catalyst-700 W-2 min, the N and S proportions are 16.8% and 4.34%, respectively, which is the highest among all of the catalysts herein, and this catalyst, therefore, exhibits the best ORR activity. The carbon–nitrogen structure is an important factor that influences ORR activity.^44^Table 4 presents the results of the convolution of the XPS peaks in Fig. 5b–f, which can be divided into components associated with quaternary-type nitrogen (401.4 eV), pyrrolic-type nitrogen (400.3 eV), and cyanide-type nitrogen (399.3 eV). This table demonstrates that the proportion of quaternary-type nitrogen of L/C catalyst-700 W-2 min is 36.1%, which exceeds those in the other samples. Wu *et al.* reported that quaternary-type nitrogen increased ORR activity.^16^ Deak *et al.* found that a high ORR activity depended on a high proportion of quaternary-type nitrogen or graphitic-type nitrogen.^45^ Moreover, S is doped in the catalyst strongly affects ORR activity, as determined by Park *et al.*^43,46–48^ In this work, L/C catalyst-700 W-2 min presents the most S content among other samples.

**Table 4:** The nitrogen-type proportions of L/C catalysts by various powers and times determined from Fig. 5b–f

Samples	Quaternary-type nitrogen (401.4 eV)	Pyrrolic-type nitrogen (400.3 eV)	Cyanide (399.3 eV)
L/C catalyst-400 W-1 min	35.6	48.9	21.8
L/C catalyst-400 W-2 min	21.9	56.3	19.5
L/C catalyst-700 W-1 min	24.6	57.2	18.2
L/C catalyst-700 W-2 min	36.1	41.2	22.7
L/C catalyst-700 W-3 min	15.2	68.2	18.2

**Fig. 6:**
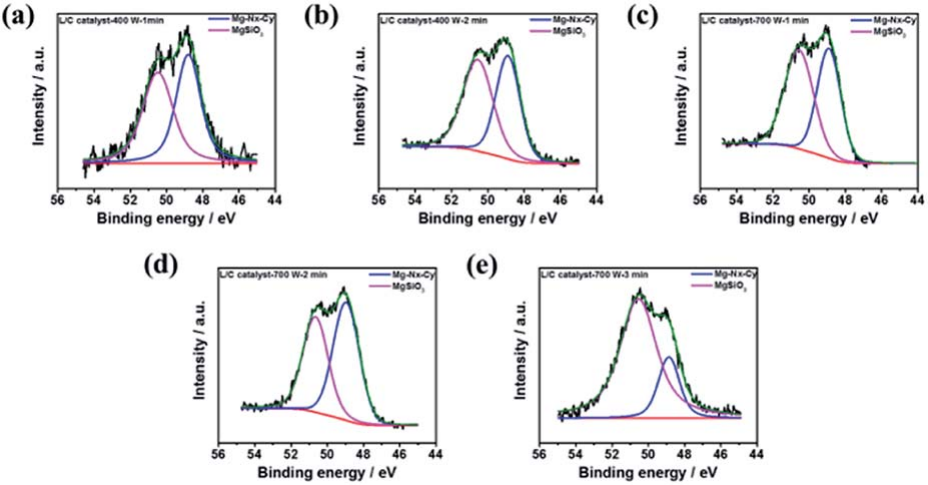
The XPS results of Mg 2p spectra of L/C catalysts at: (a) 400 W-1 min, (b) 400 W-2 min, (c) 700 W-1 min, (d) 700 W-2 min, (e) 700 W-3 min.

Besides, the Mg–N_*x*_–C_*y*_ structure can be associated with the activity of ORR. It also can be demonstrated by XPS analysis. Here, Fig. 6a to e show the deconvolution of XPS in Mg 2p. It denotes the area ratios of Mg–N_*x*_–C_*y*_ and MgSiO_3_. The results can be summarized in Table 5. It indicates the highest ratio of Mg–N_*x*_–C_*y*_ in L/C catalyst-700 W-2 min among other catalysts. It means that most amount of N_*x*_-structure is kept and is not destroyed by microwave-assisted in this power and treated time. The N_4_ structure is destroyed and converted to MgSiO_3_ by high power and long-time treatment. However, the 700 W-3 min has the lowest ratio of Mg–N_*x*_–C_*y*_.

**Table 5:** The magnesium-type proportions of L/C catalysts by various powers and times determined from Fig. 6a–e

Samples	Mg–N_*x*_–C_*y*_ (48.9 eV)	MgSiO_3_ (50.4 eV)
L/C catalyst-400 W-1 min	50.4	49.6
L/C catalyst-400 W-2 min	46.9	53.1
L/C catalyst-700 W-1 min	47.2	52.8
L/C catalyst-700 W-2 min	54.9	45.1
L/C catalyst-700 W-3 min	22.1	77.9

The results are consistent with XRD, which is shown in Fig. 3. The complete N_*x*_-structure has been proved that it can improve the ORR activity from our previous results.^17–19,44,49–51^ These results can offer powerful evidence to explain why the best ORR activity was observed in L/C catalyst-700 W-2 min.

**Fig. 7:**
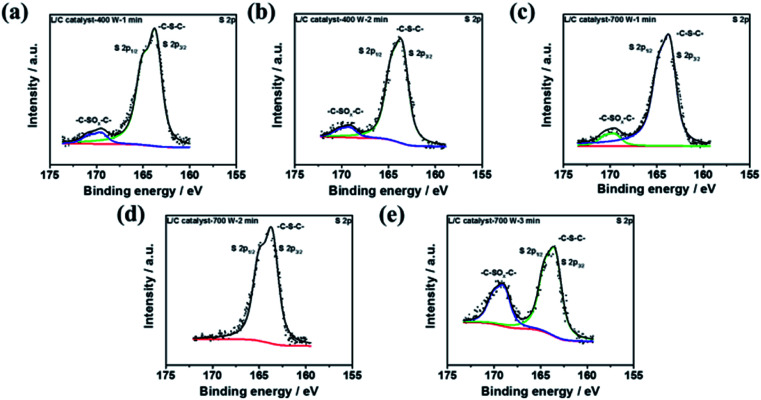
The XPS results of S 2p spectra of L/C catalysts at: (a) 400 W-1 min, (b) 400 W-2 min, (c) 700 W-1 min, (d) 700 W-2 min, (e) 700 W-3 min.

To discuss the effect of sulfur-containing structure, the XPS S 2p spectra of several L/C catalysts are shown in Fig. 7, in which the deconvolution results are shown in Table 6. L/C catalyst-700 W-2 min almost shows 100% of –C–S–C– but no –C–SO_*x*_–C–. Yang *et al.* reported sulfur-doped graphene has good oxygen reduction activity because of sulfur-doped carbon materials, breaking the electroneutrality of graphitic materials owing to the different electroneutrality between carbon and the dopant would create favorable positive charged sites for the side-on O_2_ surface adsorption. This parallel diatomic adsorption could effectively weaken the O–O bonding and facilitate the direct reduction of oxygen to OH^−^*via* a four-electron process.^52^ Moreover, Wang *et al.* indicated the cell performance improvement of Fe/N/C after doping of S. The –C–S–C– enhance the ORR activity sites of the catalyst.^53^ Li *et al.* mentioned that –C–SO_*x*_–C– is inactive for ORR but –C–S–C– not only enhances the ORR activity but also improves the ORR stability of the catalyst.^54^ Moreover, –C–SO_*x*_–C– inhibits the ability of ORR in the catalyst. According to these reasons, it can be explained why L/C catalyst-700 W-2 min shows the excellent ORR ability than other conditions due to the highest ratio –C–S–C– structure.

**Table 6:** The sulfide-type proportions of L/C catalysts by various powers and times determined from Fig. 7a–e

Samples	–C–S–C– (163.8 eV)	–C–SO_*x*_–C– (167.5–171.5 eV)
L/C catalyst-400 W-1 min	95.3	4.7
L/C catalyst-400 W-2 min	85.8	14.2
L/C catalyst-700 W-1 min	87.3	13.7
L/C catalyst-700 W-2 min	100.0	—
L/C catalyst-700 W-3 min	69.4	30.6

**Fig. 8:**
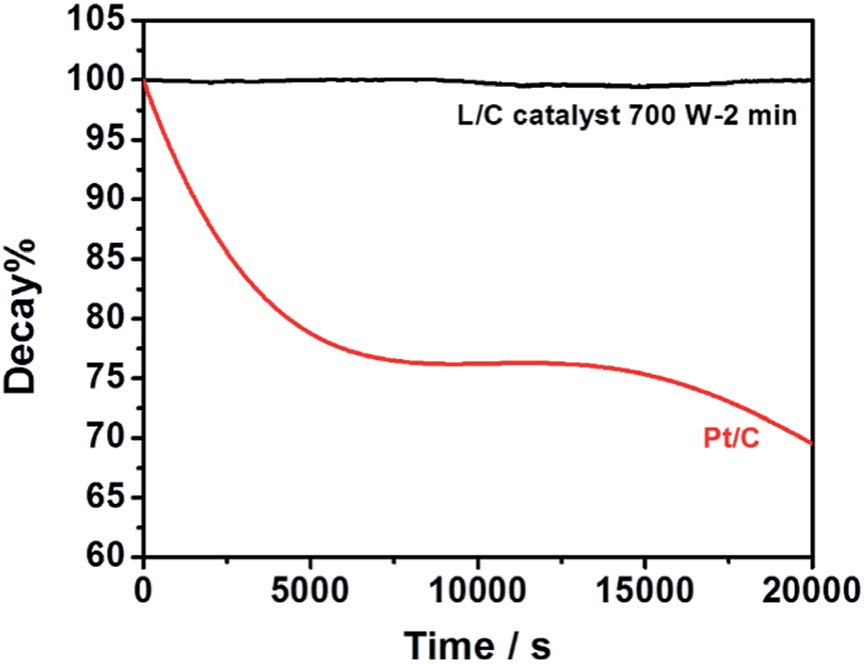
Current retentions of L/C catalyst-700 W-2 min and Pt/C kept by the constant potential of 0.76 V in O_2_-saturated 0.1 M KOH.

L/C catalyst-700 W-2 min and Pt/C in the ORR are used for the chronoamperometric measurements at 0.76 V, as shown in Fig. 8. The plots show the current retentions of L/C catalyst-700 W-2 min and Pt/C normalized to the initial current. The L/C catalyst-700 W-2 min almost shows no decay after 20 000 s – exceeding that of Pt/C. Pt/C is degraded by the blocking effect of the OH_ads_ species that are adsorbed on the Pt surface.^55^ However, the OH_ads_ species have almost no effect on L/C catalyst-700 W-2 min, leading to the excellent stability of L/C catalyst-700 W-2 min.

Accordingly, the above electrochemical and structural results are simply demonstrated to elucidate the mechanism of ORR for L/C catalyst. The quaternary-type nitrogen, Mg–N_*x*_–C_*y*_, and –C–S–C– are evaluated to promote the dominant ORR ability.

## Conclusions

In this work, microwave treated catalyst composed of *Pachira aquatica* leaves and carbon black were demonstrated to exhibit high ORR activity. This can be attributed to the combination of the nitrogen-rich Mg–N_*x*_–C_*y*_ structure and sulfur-rich containing leaves. XPS, Raman spectra and XRD results show that the ORR activity is related to the constituent elements of the leaves, including N, S, C, and Si. Superior ORR activity and long-term stability depend on high ratios of quaternary-type nitrogen and Mg–N_*x*_–C_*y*_/MgSiO_3_ and –C–S–C– species which are chiefly responsible for improving the ORR ability. However, the –C–SO_*x*_–C– is a species to inhibit ORR activity. The L/C catalyst is produced from natural N_4_-structure materials for a low-waste and non-toxic process; the microwave-assisted method is used for the cheap, facile, and fast process. The energy thus generated can truly be called “green” energy. In the future, this catalyst shall be modified and improved, enabling it to compete with the performance of Pt.

## Conflicts of interest

There are no conflicts to declare.

## Supplementary Material

## References

[cit1] Zhang W., Wang R., Wang H., Lei Z. (2010). Fuel Cells.

[cit2] Jang J.-H., Kim J., Lee Y.-H., Kim I. Y., Park M.-H., Yang C.-W., Hwang S.-J., Kwon Y.-U. (2011). Energy Environ. Sci..

[cit3] Morozan A., Jousselme B., Palacin S. (2011). Energy Environ. Sci..

[cit4] Cai Y., Ma C., Zhu Y. M., Wang J. X., Adzic R. R. (2011). Langmuir.

[cit5] Wang R., Xu C., Bi X., Ding Y. (2012). Energy Environ. Sci..

[cit6] Huang S.-Y., Ganesan P., Popov B. N. (2012). ACS Catal..

[cit7] Jasinski R. (1964). Nature.

[cit8] Pylypenko S., Mukherjee S., Olson T. S., Atanassov P. (2008). Electrochim. Acta.

[cit9] Nallathambi V., Lee J.-W., Kumaraguru S. P., Wu G., Popov B. N. (2008). J. Power Sources.

[cit10] Lefevre M., Dodelet J.-P. (2008). Electrochim. Acta.

[cit11] Deng X. Y., Wang X., Ma Z. F. F. (2008). J. Power Sources.

[cit12] Bezerra C. W. B., Zhang L., Lee K., Liu H., Marques A. L. B., Marques E. P., Wang H., Zhang J. (2008). Electrochim. Acta.

[cit13] Lefevre M., Proietti E., Jaouen F., Dodelet J. P. (2009). Science.

[cit14] Proietti E., Jaouen F., Lefevre M., Larouche N., Tian J., Herranz J., Dodelet J. P. (2011). Nat. Commun..

[cit15] Wu G., Johnston C. M., Mack N. H., Artyushkova K., Ferrandon M., Nelson M., Lezama-Pacheco J. S., Conradson S. D., More K. L., Myers D. J., Zelenay P. (2011). J. Mater. Chem..

[cit16] Wu G., More K. L., Johnston C. M., Zelenay P. (2011). Science.

[cit17] Chang S.-T., Wang C.-H., Du H.-Y., Hsu H.-C., Kang C.-M., Chen C.-C., Wu J. C. S., Yen S.-C., Huang W.-F., Chen L.-C., Lin M. C., Chen K.-H. (2012). Energy Environ. Sci..

[cit18] Huang H.-C., Shown I., Chang S.-T., Hsu H.-C., Du H.-Y., Kuo M.-C., Wong K.-T., Wang S.-F., Wang C.-H., Chen L.-C., Chen K.-H. (2012). Adv. Funct. Mater..

[cit19] Huang H.-C., Wang C.-H., Shown I., Chang S.-T., Hsu H.-C., Du H.-Y., Chen L.-C., Chen K.-H. (2013). J. Mater. Chem. A.

[cit20] Su H., Wang X.-T., Hu J.-X., Ouyang T., Xiao K., Liu Z.-Q. (2019). J. Mater. Chem. A.

[cit21] Zhang H., He J., Zhai C., Zhu M. (2019). Chin. Chem. Lett..

[cit22] Yuan Y., Wang J., Adimi S., Shen H., Thomas T., Ma R., Attfield J. P., Yang M. (2020). Nat. Mater..

[cit23] Liang Y. Y., Li Y. G., Wang H. L., Zhou J. G., Wang J., Regier T., Dai H. J. (2011). Nat. Mater..

[cit24] Liang Y. Y., Wang H. L., Zhou J. G., Li Y. G., Wang J., Regier T., Dai H. J. (2012). J. Am. Chem. Soc..

[cit25] Liang Y. Y., Wang H. L., Diao P., Chang W., Hong G. S., Li Y. G., Gong M., Xie L. M., Zhou J. G., Wang J., Regier T. Z., Wei F., Dai H. J. (2012). J. Am. Chem. Soc..

[cit26] Wang X.-T., Ouyang T., Wang L., Zhong J.-H., Ma T., Liu Z.-Q. (2019). Angew. Chem., Int. Ed..

[cit27] Wang C.-H., Yang C.-W., Lin Y.-C., Chang S.-T., Chang S. L. Y. (2015). J. Power Sources.

[cit28] Huang H.-C., Lin Y.-C., Chang S.-T., Liu C.-C., Wang K.-C., Jhong H.-P., Lee J.-F., Wang C.-H. (2017). J. Mater. Chem. A.

[cit29] Huang H.-C., Su C.-Y., Wang K.-C., Chen H.-Y., Chang Y.-C., Chen Y.-L., Wu K. C. W., Wang C.-H. (2019). ACS Sustainable Chem. Eng..

[cit30] Jhong H.-P., Chang S.-T., Huang H.-C., Wang K.-C., Lee J.-F., Yasuzawa M., Wang C.-H. (2019). Catal. Sci. Technol..

[cit31] Wang K.-C., Huang H.-C., Chang S.-T., Wu C.-H., Yamanaka I., Lee J.-F., Wang C.-H. (2019). ACS Sustainable Chem. Eng..

[cit32] Pan F., Cao Z., Zhao Q., Liang H., Zhang J. (2014). J. Power Sources.

[cit33] Gao S., Wei X., Fan H., Li L., Geng K., Wang J. (2015). Nano Energy.

[cit34] Razmjooei F., Singh K. P., Yu J.-S. (2016). Catal. Today.

[cit35] Gao S., Geng K., Liu H., Wei X., Zhang M., Wang P., Wang J. (2015). Energy Environ. Sci..

[cit36] Zhu B., Qiu K., Shang C., Guo Z. (2015). J. Mater. Chem. A.

[cit37] Zhang L. Y., Zhou Z., Liu Z., Li C. M. (2016). ChemElectroChem.

[cit38] Huang Y., Wu D., Cao D., Cheng D. (2018). Int. J. Hydrogen Energy.

[cit39] Bajpai S. K., Jain A. (2012). Water.

[cit40] Ungar T., Gubicza J., Ribarik G., Pantea C., Zerda T. W. (2002). Carbon.

[cit41] You C., Liao S., Li H., Hou S., Peng H., Zeng X., Liu F., Zheng R., Fu Z., Li Y. (2014). Carbon.

[cit42] Xu L., Pan G., Liang X. (2014). RSC Adv..

[cit43] Chu J. P., Liu T.-Y., Li C.-L., Wang C.-H., Jang J. S. C., Chen M.-J., Chang S.-H., Huang W.-C. (2014). Thin Solid Films.

[cit44] Chang S.-T., Huang H.-C., Wang H.-C., Hsu H.-C., Lee J.-F., Wang C.-H. (2014). Int. J. Hydrogen Energy.

[cit45] von Deak D., Singh D., Biddinger E. J., King J. C., Bayram B., Miller J. T., Ozkan U. S. (2012). J. Catal..

[cit46] Xu L., Pan G., Liang X., Luo G., Zou C., Chen G. (2014). J. Energy Chem..

[cit47] Park J. E., Jang Y. J., Kim Y. J., Song M. S., Yoon S., Kim D. H., Kim S. J. (2014). Phys. Chem. Chem. Phys..

[cit48] Inamdar S., Choi H.-S., Wang P., Song M. Y., Yu J.-S. (2013). Electrochem. Commun..

[cit49] Wang C.-H., Huang H.-C., Chang S.-T., Lin Y.-C., Huang M.-F. (2014). RSC Adv..

[cit50] Chang S.-T., Hsu H.-C., Huang H.-C., Wang C.-H., Du H.-Y., Chen L.-C., Lee J.-F., Chen K.-H. (2012). Int. J. Hydrogen Energy.

[cit51] Wang C.-H., Chang S.-T., Hsu H.-C., Du H.-Y., Wu J. C.-S., Chen L.-C., Chen K.-H. (2011). Diamond Relat. Mater..

[cit52] Yang Z., Yao Z., Li G., Fang G., Nie H., Liu Z., Zhou X., Chen X. a., Huang S. (2012). ACS Nano.

[cit53] Wang Y. C., Lai Y. J., Song L., Zhou Z. Y., Liu J. G., Wang Q., Yang X. D., Chen C., Shi W., Zheng Y. P., Rauf M., Sun S. G. (2015). Angew. Chem..

[cit54] Li J. S., Li S. L., Tang Y. J., Li K., Zhou L., Kong N., Lan Y. Q., Bao J. C., Dai Z. H. (2014). Sci. Rep..

[cit55] Ramaswamy N., Mukerjee S. (2012). Adv. Phys. Chem..

